# The combined signatures of telomere and immune cell landscape provide a prognostic and therapeutic biomarker in glioma

**DOI:** 10.3389/fimmu.2023.1220100

**Published:** 2023-08-17

**Authors:** Xu Han, Zihan Yan, Kaiyu Fan, Xueyi Guan, Bohan Hu, Xiang Li, Yunwei Ou, Bing Cui, Lingxuan An, Yaohua Zhang, Jian Gong

**Affiliations:** ^1^ Department of Neurosurgery, Beijing Tiantan Hospital, Capital Medical University, Beijing, China; ^2^ Beijing Institute of Brain Disorders, Laboratory of Brain Disorders, Ministry of Science and Technology, Collaborative Innovation Center for Brain Disorders, Capital Medical University, Beijing, China; ^3^ Department of General, Visceral, and Transplant Surgery, Ludwig-Maximilians-University Munich, Munich, Germany; ^4^ Beijing Neurosurgical Institute, Capital Medical University, Beijing, China

**Keywords:** telomere related genes, glioma, tumor microenvironment, prognosis, immune response

## Abstract

**Background:**

Gliomas, the most prevalent primary malignant tumors of the central nervous system in adults, exhibit slow growth in lower-grade gliomas (LGG). However, the majority of LGG cases progress to high-grade gliomas, posing challenges for prognostication. The tumor microenvironment (TME), characterized by telomere-related genes and immune cell infiltration, strongly influences glioma growth and therapeutic response. Therefore, our objective was to develop a Telomere-TME (TM-TME) classifier that integrates telomere-related genes and immune cell landscape to assess prognosis and therapeutic response in glioma.

**Methods:**

This study encompassed LGG patients from the TCGA and CCGA databases. TM score and TME score were derived from the expression signatures of telomere-related genes and the presence of immune cells in LGG, respectively. The TM-TME classifier was established by combining TM and TME scores to effectively predict prognosis. Subsequently, we conducted Kaplan-Meier survival estimation, univariate Cox regression analysis, and receiver operating characteristic curves to validate the prognostic prediction capacity of the TM-TME classifier across multiple cohorts. Gene Ontology (GO) analysis, biological processes, and proteomaps were performed to annotate the functional aspects of each subgroup and visualize the cellular signaling pathways.

**Results:**

The TM_low+TME_high subgroup exhibited superior prognosis and therapeutic response compared to other subgroups (P<0.001). This finding could be attributed to distinct tumor somatic mutations and cancer cellular signaling pathways. GO analysis indicated that the TM_low+TME_high subgroup is associated with the neuronal system and modulation of chemical synaptic transmission. Conversely, the TM_high+TME_low subgroup showed a strong association with cell cycle and DNA metabolic processes. Furthermore, the classifier significantly differentiated overall survival in the TCGA LGG cohort and served as an independent prognostic factor for LGG patients in both the TCGA cohort (P<0.001) and the CGGA cohort (P<0.001).

**Conclusion:**

Overall, our findings underscore the significance of the TM-TME classifier in predicting prognosis and immune therapeutic response in glioma, shedding light on the complex immune landscape within each subgroup. Additionally, our results suggest the potential of integrating risk stratification with precision therapy for LGG.

## 1 Introduction

Glioma, which accounts for approximately 80% of all malignant brain tumors, represents one of the most commonly observed primary brain tumors in clinical practice (1). The 2016 World Health Organization (WHO) revision classifies glioma into four grades. Grades II and III are collectively referred to as diffuse lower-grade gliomas (LGG), while grade IV is known as glioblastoma (GBM) (2). GBM is a fatal tumor with a median overall survival (OS) of merely 15 months. In contrast, patients diagnosed with WHO II and WHO III gliomas have median OS durations of 78.1 and 37.6 months, respectively (3). Despite advancements in diagnostic and therapeutic outcomes, a subset of LGG patients may experience disease progression, leading to unfavorable therapeutic responses and a worsened prognosis.

The conventional treatment modalities for LGG encompass surgical intervention, radiotherapy, and chemotherapy. However, the emergence of immunotherapy has introduced a potential revolution in clinical management and improved survival rates for LGG patients. Nonetheless, its efficacy remains limited to a small subset of individuals. Currently, predictive biomarkers utilized in clinical practice include O6-methylguanine DNA methyltransferase (MGMT) promoter methylation, 1p/19q codeletion, and isocitrate dehydrogenase (IDH) mutation, among others (4, 5). Nevertheless, the existing pool of investigated biomarkers fails to provide sufficient accuracy for prognosis prediction and optimal treatment selection.

Telomeres, the repeated TTAGGG DNA sequences located at chromosome ends, are associated with the shelterin complex (6). These specialized structures are essential for chromosome stability, and aberrations in telomeres have been implicated in numerous diseases, including various forms of cancer (7). A 2017 Mendelian randomization study analyzing 16 telomere length-related single nucleotide polymorphisms (SNPs) from 130 genome-wide association studies (GWAS) revealed that genetically longer telomeres are associated with an elevated risk of several cancers, including lung cancer, ovarian cancer, neuroblastoma, and glioma (8). The primary regulation of telomere length is attributed to the telomerase complex, comprising an RNA template and the telomerase reverse transcriptase (TERT) enzyme (9). Notably, TERT exerts significant influence on angiogenesis, invasion, epithelial-mesenchymal transformation (EMT), inflammation, immunosuppression, and other critical gene expression profiles, even in a telomere-independent manner. These TERT-mediated activities may profoundly affect the dynamics and homeostasis of the tumor microenvironment (TME) (10–12). Several studies have validated the association between TERT promoter mutations and the prognosis of glioma patients. In particular, individuals with TERT promoter mutations demonstrated a more favorable prognosis compared to those with wildtype TERT promoter, specifically in grade II or III oligodendroglioma, 1p/19q-codeleted patients, and IDH-mutant cases (13, 14).

In the present study, we leveraged the features of telomeres and immune cells to establish Telomere (TM) and TME scores, respectively. Recognizing the telomere-mediated regulation of TME and the implications for prognosis and immunotherapy, we developed an integrated TM-TME classifier utilizing these scores to effectively predict prognosis and immunotherapy response. Remarkably, distinct patient subgroups within the LGG cohort exhibited varying prognostic outcomes, somatic mutation (SM) landscapes, responses to therapy, and enriched pathways. These findings hold promising implications for the improvement of clinical disease management.

## 2 Methods

### 2.1 Source of the data

The training set for this study comprised the mRNA expression profiles and corresponding clinical data of the TCGA LGG cohort, which were obtained from the UCSC Xena database (https://xena.ucsc.edu/). To validate the findings, we also acquired the mRNAseq_693 dataset from the CGGA database, serving as the confirmation set. Our analysis focused exclusively on LGG patients with WHO grades II and III, utilizing the aforementioned datasets. To visualize the TM scores within each cell, we obtained two public single-cell RNA sequencing (scRNA-seq) cohorts, namely GSE70630 and GSE89567, from the Gene Expression Omnibus repository (GEO) (15), specifically the mRNAseq_693 data set. The analysis focused exclusively on LGG patients classified as WHO grades II and III in the aforementioned datasets. Moreover, two publicly available single-cell RNA sequencing (scRNA-seq) cohorts, namely GSE70630 and GSE89567, were retrieved from the GEO (Gene Expression Omnibus repository) to enable the visualization of TM scores at the cellular level (16, 17). Subsequently, the bulk RNA sequencing data underwent a log2(TPM+1) transformation for further analyses.

### 2.2 Identification of prognostic telomere-associated genes and TME cells

We utilized the URL of http://www.cancertelsys.org/telnet/to obtain the genes associated with telomeres (18). In order to identify prognostic-associated telomere genes in the TCGA-LGG dataset, we performed univariate Cox regression analysis and least absolute shrinkage and selection operator (LASSO) regression analysis on 2093 telomere-associated genes using the bootstrap approach (18, 19). We utilized the URL of http://www.cancertelsys.org/telnet/to obtain the genes associated with telomeres (18). In order to identify prognostic-associated telomere genes in the TCGA-LGG dataset, we performed univariate Cox regression analysis and least absolute shrinkage and selection operator (lasso) regression analysis on 2093 telomere-associated genes using the bootstrap approach (20, 21). The TCGA LGG cohort incorporates the CIBERSORT algorithm, which estimates the composition of 22 immune cells in distinct tissues based on gene expression signatures for TME cells (22, 23). The TCGA LGG cohort incorporates the CIBERSORT algorithm, which estimates the composition of 22 immune cells in distinct tissues based on gene expression signatures for TME cells.

### 2.3 Construction of TM score, TME score, and TM-TME classifier

The establishment of the TM and TME scores was based on the 18 telomere-associated genes derived from the TCGA LGG cohort and the coefficients (Coef) of five immune cells for multivariate Cox regression analysis (24). To enhance the accuracy of both the TM and TME models, we randomly selected 1000 samples from the entire LGG sample pool and conducted multivariate Cox analysis on each sample (25). Moreover, we obtained the standard deviation (SD) values of the Coef for each gene and cell. The weights in the respective models were determined by the ratio of the Coef to the SD values. In summary, the following formula was employed to calculate the TM score:


$$TM\;score=\sum_{i=1}^{18}\frac{Coef_{i}}{SD_{i}}*\;exp\left(gene_{i}\right)$$

Similarly, the TME score calculating formula is:


$$TME\;score=-\sum_{j=1}^{5}\frac{Coef_{j}}{SD_{j}}*\;fra\left(cell_{j}\right)$$

Where exp(genei) denotes the gene i’s expression level and fra(cellj) denotes the cell j’s fraction. Using the median value of each dataset’s TM and TME scores, the TM-TME classifier was then constructed. Samples in each cohort were divided into “TM_low+TME_high (TM_L+TME_H)”, “TM_high+TME_low (TM_H+TME_L)”, and “Mixed” [TM_low+TME_low (TM_L+TME_L), TM_high+TME_high (TM_H+TME_H)] groups. The “timeROC” package was utilized for assessing the prognostic prediction power of the TM-TME classifier using receiver operating characteristic (ROC) curves (26, 27).

### 2.4 Visualization of TM score at the single-cell level

To analyze the single-cell data, we employed Seurat objects designed for scRNA-seq gene expression matrix, enabling clustering analysis, annotation, and visualization (28, 29). Transcriptomes expressing between 200 and 7000 genes were retained. To reduce dimensionality, a principal component (PC) analysis was conducted on the top 3000 genes exhibiting the highest variability. Subsequently, t-SNE (t-distributed stochastic neighbor embedding) was applied for visualizing the single-cell data. Cell type annotations were derived using cell type markers reported in the published literature (30, 31). The aforementioned formula related to the TM model was utilized to compute the TM score for each individual cell.

In addition, we utilized The Tumor Immune Single-Cell Hub (TISCH; http://tisch.comp-genomics.org) (32, 33), an extensive online resource providing specific single-cell RNA-seq data related to the tumor microenvironment (TME), to systematically investigate the diverse composition of the TME across distinct datasets and cell types.

### 2.5 Weighted gene co-expression network analysis, enrichment analysis, and construction of the interacted network

The identification of the gene module influencing the TM-TME classifier was achieved using WGCNA (34, 35). WGCNA analysis was performed on the TCGA LGG expression profile, selecting the top 5000 genes based on their median absolute deviations. To discover potential hub genes, the key module exhibiting the strongest association with the subgroups was determined. Subsequent evaluation of hub genes within the co-expression module of highest significance was conducted using Metascape (http://metascape.org) (36), a gene function enrichment and categorization tool. To uncover interconnected hub genes, the MCODE algorithm was employed. Each MCODE network was assigned a distinct color to represent the close interacting associations between molecules. Additionally, a list of differentially expressed proteins was inputted into an internet tool (https://proteomaps.net/) to generate proteomaps (37).

### 2.6 SM and immunotherapy response

The mutation annotation format (MAF) data pertaining to the TCGA LGG cohort could be accessed through the TCGA database. In order to compare the SM status within different subgroups of the TM-TME classifier, we generated waterfall diagrams using the “maftools” package to illustrate the mutations in the top 20 genes (38, 39). Moreover, the tumor mutational burden (TMB) of each LGG sample was calculated by determining the total number of somatic mutations (SMs) per million bases in the tumor genome, after excluding germ-line mutations. To predict the clinical response to immune checkpoint blockade (ICB) in melanoma and other tumor patients, the TIDE web platform incorporates relevant ICB trials published in the literature (40, 41).

### 2.7 Total RNA extraction and quantitative real-time polymerase chain reaction

We employed quantitative real-time polymerase chain reaction (qRT-PCR) to investigate the expression patterns of 18 selected pivotal genes in both LGG tissues and their corresponding peritumoral tissues, which were obtained from a cohort of five patients who underwent surgical intervention. Total RNA was extracted using TRIZOL reagent (Life technologies, USA), followed by reverse transcription reactions performed with a qPCR RT Kit (Yeason, China). Subsequently, qRT-PCR was conducted on a LightCycler 480 SYBR Green I instrument (Roche, Manheim, Germany) to determine the expression levels of the target genes (42). The forward and reverse primers for the 18 pivotal genes and GAPDH were provided in **Supplementary Table S3**. To normalize the gene expression, the expression of GAPDH was utilized as a reference, and the relative expression levels of the 18 key mRNAs were calculated using the 2−ΔΔCt method.

### 2.8 Western blotting, immunohistochemical, and immunofluorescence staining

Tumor and peritumoral specimens were lysed by RIPA buffer (Solarbio, Beijing, China) supplemented with protease and phosphatase inhibitors. Subsequently, the lysates were subjected to SDS-PAGE separation and transferred onto PVDF membranes. Immunoblotting was performed using antibodies against WEE1 (CST, 13084) and β-actin (proteintech, HRP-60008).

For immunohistochemical and immunofluorescence analyses, tumor and corresponding peritumoral tissues were obtained from three patients. Paraffin-embedded tissues were sectioned and subjected to heat treatment in sodium citrate buffer. Blocking of the sections was accomplished using goat serum, followed by overnight incubation with primary antibodies at 4°C. Subsequently, a secondary antibody conjugated with horseradish peroxidase was applied, and 3’-diaminobenzidine was utilized for sample visualization. Hematoxylin was used for counterstaining, and the sections were examined under a microscope.

### 2.9 Statistical analysis

In the current study, version 4.1.2 of the R statistical software package was utilized for all statistical analyses. The examination of relationships between variables was conducted using both Pearson and Spearman correlation methods. To compare different subgroups, nonparametric tests including the Wilcoxon rank sum test and Kruskal-Wallis rank sum test were employed. The significance level was set at p< 0.05, and statistical significance was denoted by “*”, “**”, and “***” for p-values less than 0.05, 0.01, and 0.001, respectively.

## 3 Results

### 3.1 Construction of TM and TME scores

**Figure 1:**
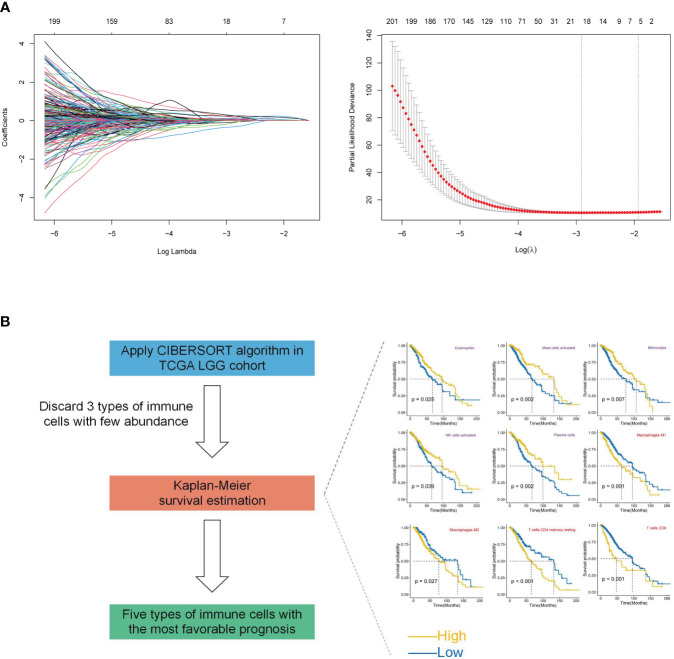
Construction of TM and TME scores. **(A)** Lasso regression analysis was performed for selected 1198 TM genes in the TCGA LGG cohort. Ultimately, the TM score was developed using 18 telomere-associated genes. **(B)** The CIBERSORT method was utilized to determine the abundance of 22 different types of immune cells in the TCGA LGG cohort. Kaplan-Meier (KM) survival curve analysis was performed for the remaining types of immune cells after excluding 3 types of immune cells with low levels. Only the types of immune cells whose p-values were< 0.05 were presented in KM overall survival curves shown on the right-hand side (favorable and unfavorable prognostic factors are respectively denoted by the colors purple and red). The TME score was determined using a combination of activated NK cells, plasma cells, monocytes, mast cells, and eosinophils.

**Figure 2:**
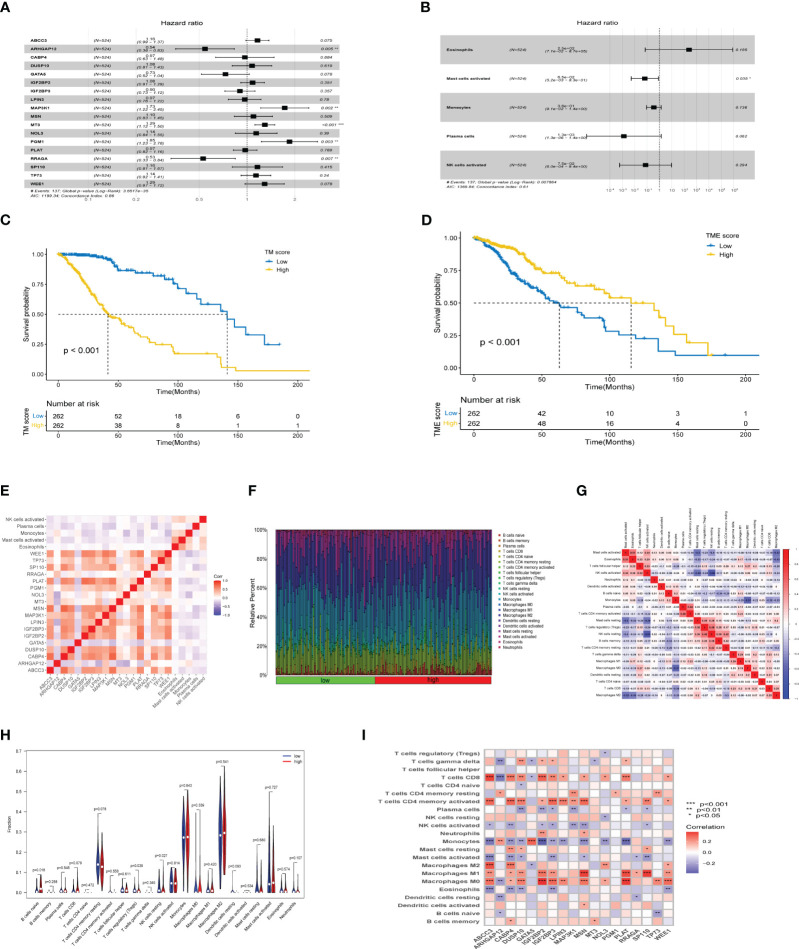
TM and TME score development and performance in LGG. **(A, B)** The multivariate cox regression-based forest plot showed 18 telomere-associated genes and 5 TME cells linked to overall survival (OS). **(C, D)** Kaplan-Meier survival curves for subgroups characterized by high and low TM and TME scores. **(E)** Heatmap showing the spearman correlation matrix of the telomere-associated genes and TME cells. The positive, negative, and insignificant correlations are denoted respectively by the colors red, blue, and blank. **(F)** The ratio of infiltrating immune cells categorized by their risk score. **(G)** Correlation of immune cells. **(H)** Differences in immune cell composition between high-risk group and low-risk group. **(I)** Correlation between 18 telomere related genes and immune cells. *p<0.05, **p<0.01, ***p<0.001.

An integrated approach was employed in the TCGA LGG cohort to estimate the telomere and immune cell status of patients. Initially, univariate cox analysis, lasso regression analysis of genes related to telomere maintenance (TM genes), and KM OS estimation of immune cells were conducted (**Figures 1A, B**). The results of the univariate cox analysis yielded 1198 TM genes, which are documented in **Supplementary Table S1**. Subsequently, the TM and tumor microenvironment (TME) scores were derived, comprising 18 TM genes and five distinct immune cell types, respectively. **Supplementary Table S2** presents a comprehensive overview of these scores. To assess the prognostic significance, multivariate analysis was performed separately for the 18 TM genes and the five immune cell types (**Figures 2A, B**). Additionally, the interplay between the 18 telomere-associated genes and the five TME cells in the TCGA LGG set was explored and visualized in **Figure 2E**. Furthermore, KM survival curves demonstrated that patients with low TM scores exhibited superior survival outcomes compared to those with high TM scores, whereas the opposite trend was observed for the TME score (**Figures 2C, D**).

### 3.2 Immune activate with different risk score in TM score

**Figure 3:**
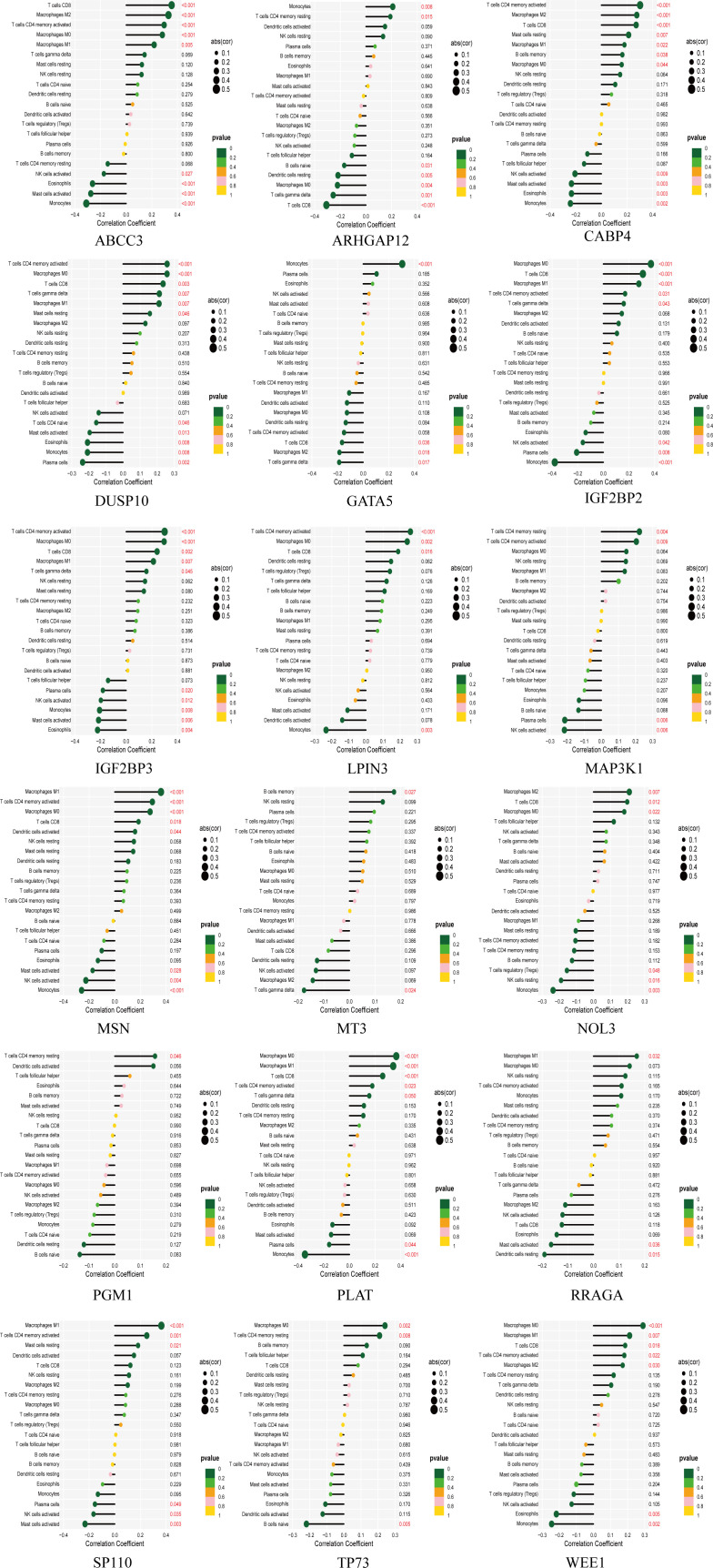
The lollipop chart showing the correlation between 18 genes and immune cells in detail.

The tumor microenvironment plays a critical role in both tumorigenesis and the efficacy of immunotherapeutic interventions. To gain a deeper understanding, we conducted further investigations into the TME milieu of patients with low-grade glioma (LGG) who were classified into high and low risk groups based on the TM score, using the CIBERSORT algorithm. Initially, we arranged the LGG patients in ascending order according to their TM risk scores, visualizing the distribution of various immune cell types based on their respective risk scores (**Figure 2F**). The interrelationships among immune cells in LGG patients (**Figure 2G**) can provide valuable insights into the immune microenvironment specific to certain tumor types. Subsequently, we observed a higher prevalence of T cells CD4 memory resting, Monocytes, and Macrophages M2 within the immune cell composition of LGG patients, leading us to hypothesize that these three cell types might exert influence on patients’ prognoses, a supposition supported by previous investigations (**Figure 2H**). Notably, the infiltration of multiple immune cell populations correlates closely with the 18 selected gene signatures employed in constructing the TM score model (**Figure 2I**). Lastly, we employed a lollipop chart to provide a detailed representation of the associations between genes and immune cell infiltrations (**Figure 3**).

### 3.3 Single-cell analysis of LGG based on TM score

**Figure 4:**
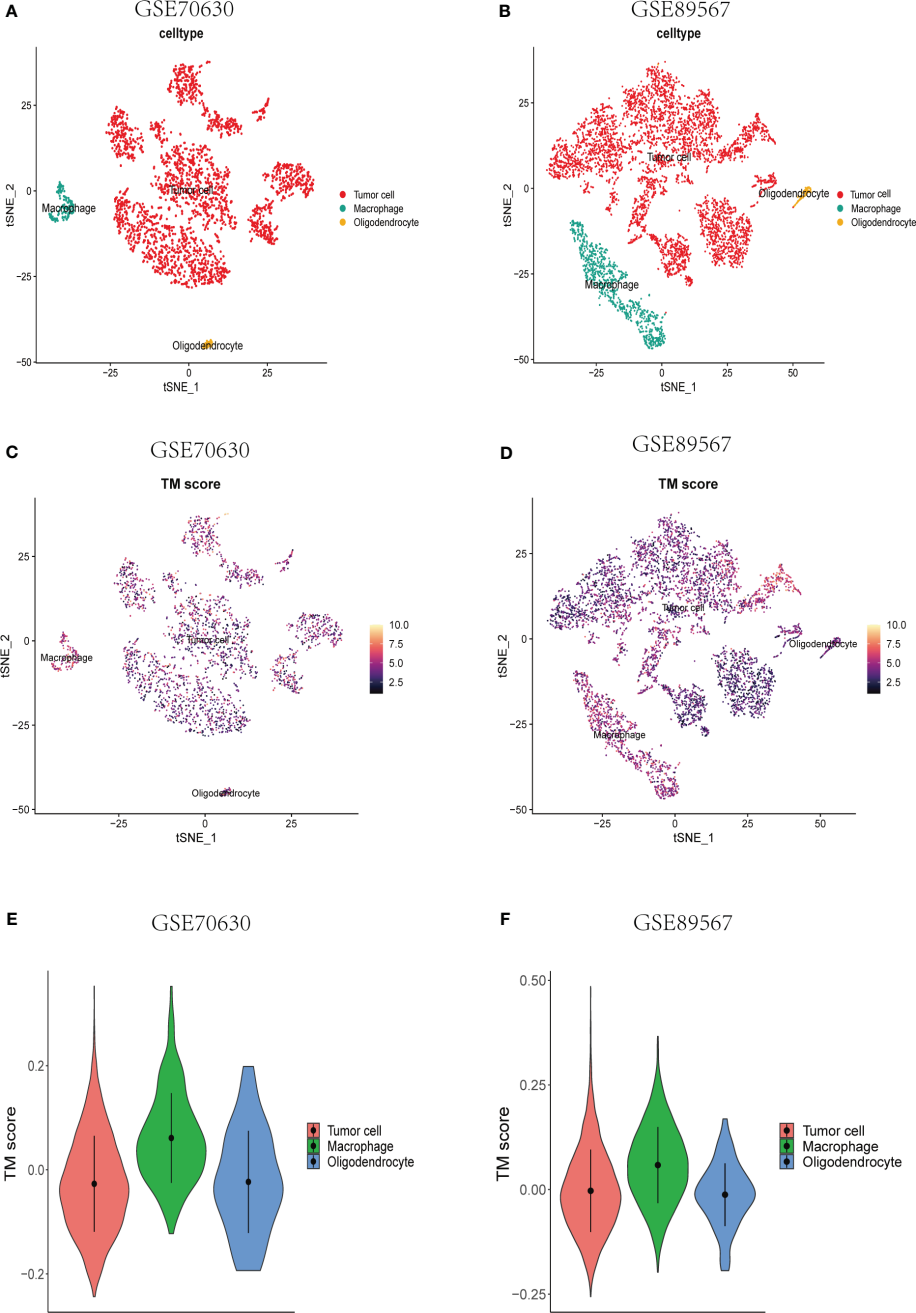
Single-cell analyses of the correlation between TM score and various types of cells at the single-cell level. **(A, B)** A t-SNE plot consisting of all cells from the different data sets. Annotation of the cells was done using the established gene markers. **(C, D)** A t-SNE plot depicting all single cells, each colored according to the TM score from the two datasets, respectively. **(E, F)** TM score comparisons across tumor cells, macrophages, and oligodendrocytes.

To investigate the TM score within the single-cell transcriptomic landscape of lower-grade glioma (LGG), we performed t-SNE analysis on single-cell datasets derived from two different sources (**Supplementary Figures S1A, B**). By assessing the elevated expression levels of gene sets corresponding to specific cell type markers, namely PTPRZ1 for tumor cells, MOBP for oligodendrocytes, and CSF1R for macrophages (**Supplementary Figures S1C, D**), we successfully classified the cells. Subsequently, we computed the TM scores individually for each cell type (**Figures 4A–D**). Notably, the macrophages exhibited considerably higher TM scores compared to both the tumor cells and oligodendrocytes (**Figures 4E, F**), thus affirming a strong association between telomere and the immune cells of the tumor microenvironment (TME) at the single-cell level.

### 3.4 Correlation analysis of 17 telomere related genes and tumor immune microenvironment at the single-cell level

**Figure 5:**
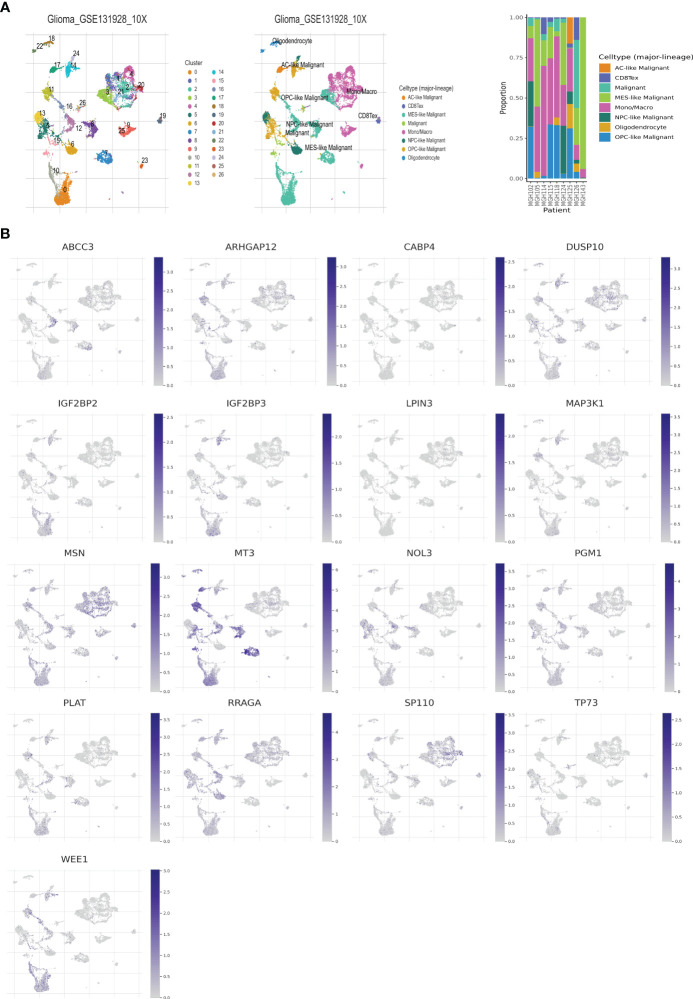
Telomere related genes expression in LGG TME-associated cells. **(A)** GSE131928 annotation of all cell types and percentage of each cell type. **(B)** Percentages of 17 telomere related genes in LGG.

**Figure 6:**
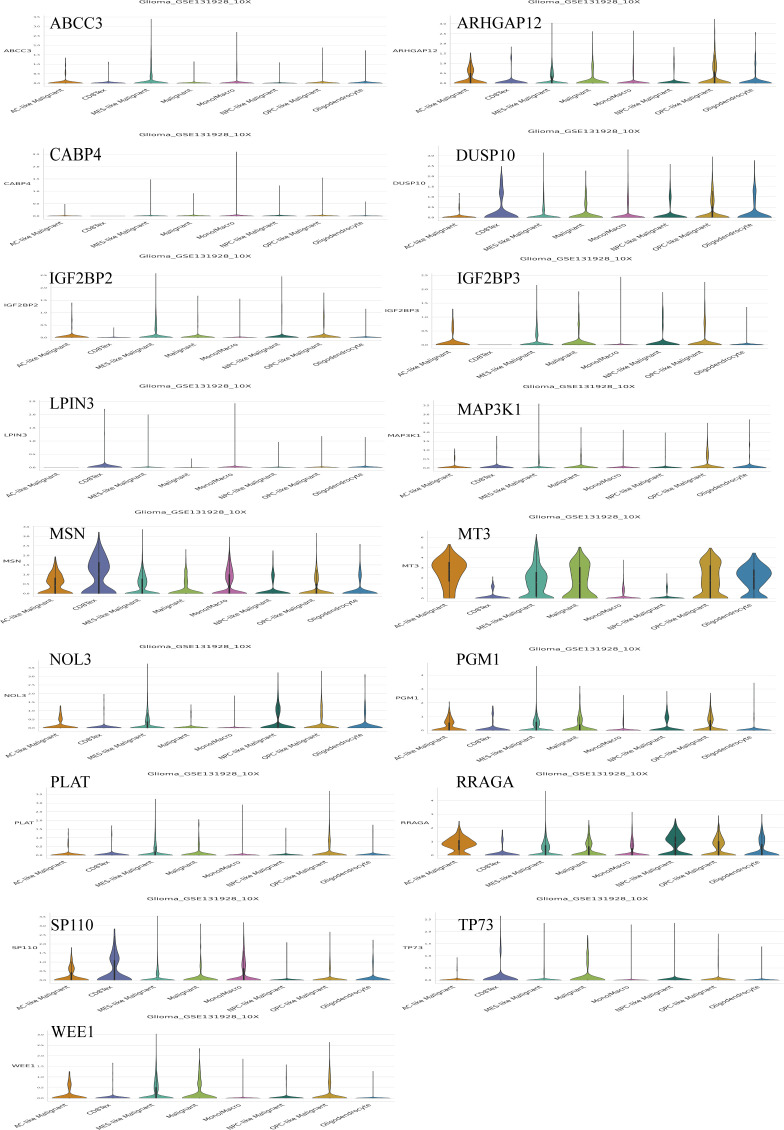
Expressions of 17 telomere related genes in LGG.

The single-cell dataset Glioma_GSE131928_10X was acquired from the TISCH database for the purpose of investigating the 17 selected genes (GATA5, which is not present in the dataset), within the TME (43). Within the GSE131928 dataset, a total of 27 cell clusters and 8 distinct cell types were identified, including malignant cells, monocytes/macrophages, oligodendrocytes, exhausted CD8+ T cells (referred to as CD8+Tex), and others. **Figure 5A** illustrates the distribution and abundance of the various cell types. AC-like malignant cells and CD8+Tex cells were found to predominantly express MSN, whereas MT3 exhibited predominant expression in malignant cells, AC-like malignant cells, MES-like malignant cells, OPC-like malignant cells, and oligodendrocytes, with minimal expression observed in NPC-like malignant cells and immune cells, CD8+Tex (**Figures 5B**, **6**).

### 3.5 TM-TME was an independent prognostic indicator for the LGG cohort

**Figure 7:**
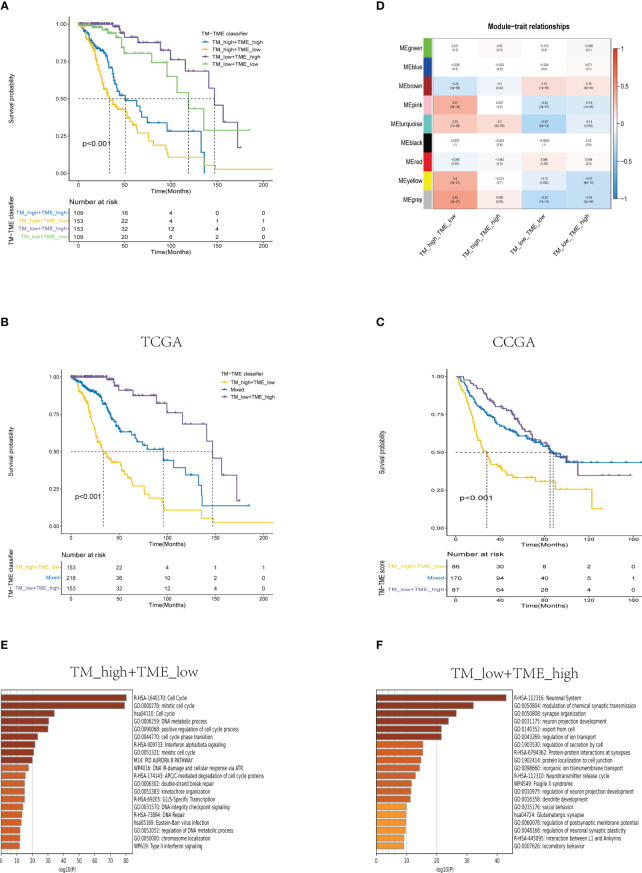
Analysis of the prognostic significance and enrichment of the TM-TME classifier. **(A)** Kaplan-Meier overall survival curves of the TCGA LGG tumors classified into four diverse subgroups as per the TM-TME classifier. **(B, C)** KM-OS curves of the training dataset (TCGA LGG cohort) and validation set (CGGA LGG cohort) based on the TM-TME classifier. **(D)** A heatmap depicting the association between the subgroups of the TM-TME classifier and the module eigengenes. The Pearson correlation coefficients as well as the P values are displayed in each cell. **(E, F)** Top 20 pathways enriched in TM_H+TME_L subgroup and TM_L+TME_H subgroup, respectively.

Based on the aforementioned findings, we developed a TM-TME classifier by utilizing the median value of TM and TME scores for each dataset. To evaluate the prognostic prediction power of the TM-TME classifier, KM OS curves were employed. The patient cohort was categorized into four subgroups based on the TM-TME classifier: TM_L+TME_H, TM_L+TME_L, TM_H+TME_H, and TM_H+TME_L. Notably, the TM-TME classifier demonstrated statistically significant prognostic implications in the TCGA LGG cohort (**Figure 7A**). It was observed that the prognostic significance is heavily influenced by both TM and TME scores. In light of the marginal prognostic disparities between TM_L+TME_L and TM_H+TME_H subgroups, a merged subgroup comprising these two subgroups was created. Consequently, the patients were divided into three subgroups: TM_L+TME_H, Mixed subgroup, and TM_H+TME_L. The novel TM-TME classifier exhibited a considerable distinction in the OS of the TCGA LGG cohort (**Figure 7B**). Optimal prognostic outcomes were noted in the TM_L+TME_H subgroup, followed by the Mixed subgroup, whereas the TM_H+TME_L subgroup displayed the poorest prognosis. Similar findings were observed in the CGGA LGG cohort (**Figure 7C**). To identify the gene module associated with the TM-TME classifier, we performed WGCNA (**Supplementary Figures S2A–C**). The key modules representing the TM_H+TME_L subgroup were the yellow and pink modules (**Figure 7D**). Similarly, we selected the brown module as the representative key module for the TM_L+TME_H subgroup. Subsequently, functional enrichment analysis was conducted using Metascape, applying a cutoff of min overlap = 3, p-value cutoff = 0.01, and min enrichment = 1.5. This analysis revealed 20 enriched pathways in the TM_H+TME_L and TM_L+TME_H subgroups (**Figures 7E, F**). The TM_H+TME_L subgroup was primarily associated with the cell cycle and DNA metabolic process, while the TM_L+TME_H subgroup exhibited a stronger association with the neuronal system and modulation of chemical synaptic transmission. For the identification of protein complexes, we utilized MCODE with the following criteria: physical score > 0.132, min network size = 3, max network size = 500, and databases as physical core (**Supplementary Figures S2D–G**).

**Figure 8:**
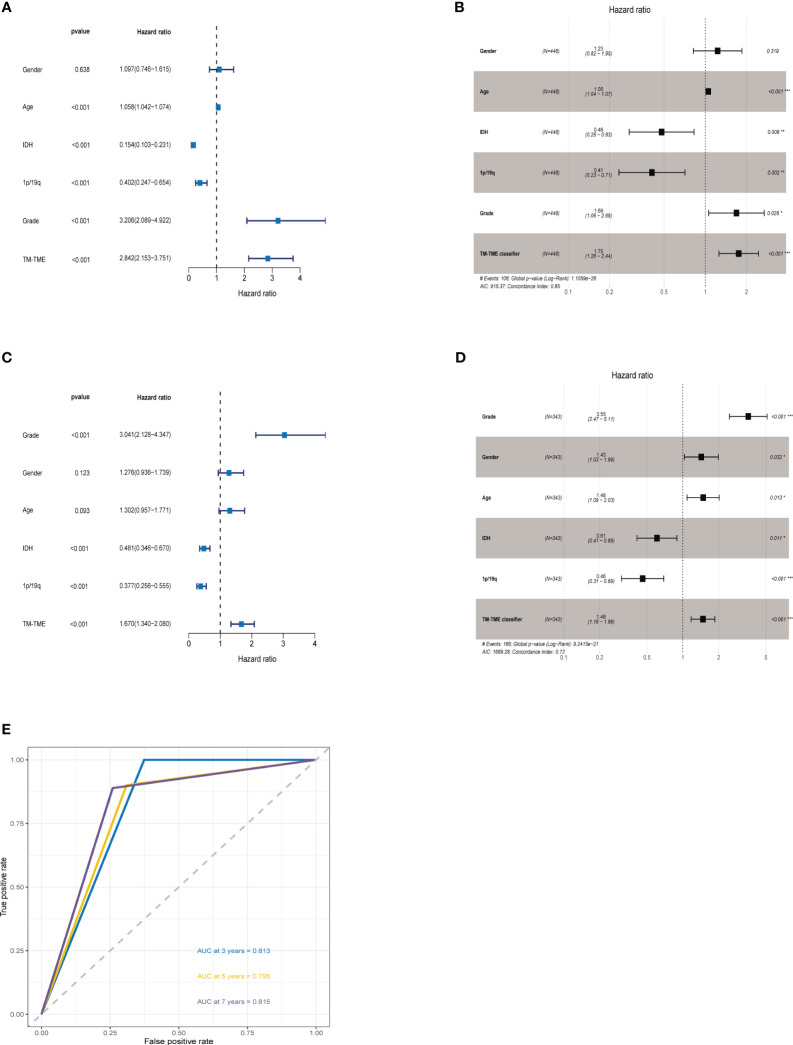
TM-TME classifier relationships with clinical characteristics in LGG. **(A, B)** Univariate and Multivariate Cox analyses of clinical characteristics and TM-TME classifier in TCGA LGG cohort. **(C, D)** Univariate and Multivariate Cox analysis of clinical characteristics and TM-TME classifier in CGGA LGG cohort. **(E)** OS-ROC curves over 3, 5, and 7 years on the basis of the TM-TME classifier in the TCGA LGG cohort.

The ROC analysis demonstrated that the TM-TME classifier effectively predicted overall survival (OS) at 3, 5, and 7 years, yielding area under the curve (AUC) values of 0.813, 0.795, and 0.815, respectively (**Figure 8E**). To investigate the comprehensive prognostic significance of the TM-TME classifier, we performed univariate and multivariate Cox regression analyses in the TCGA LGG cohort. These analyses revealed that the TM-TME classifier served as an independent unfavorable prognostic factor (**Figures 8A, B**). Additionally, this finding was validated in the CGGA LGG cohort (**Figures 8C, D**). Furthermore, we generated KM OS curves for the TM-TME classifier across various TCGA LGG clinical subtypes (**Supplementary Figure S3**). These curves demonstrated the effective prognostic prediction of the TM-TME classifier in the majority of TCGA LGG clinical subtypes. Therefore, the TM-TME classifier exhibits broad applicability for predicting prognosis in LGG.

### 3.6 Distinct SM landscapes across TM-TME subgroups

**Figure 9:**
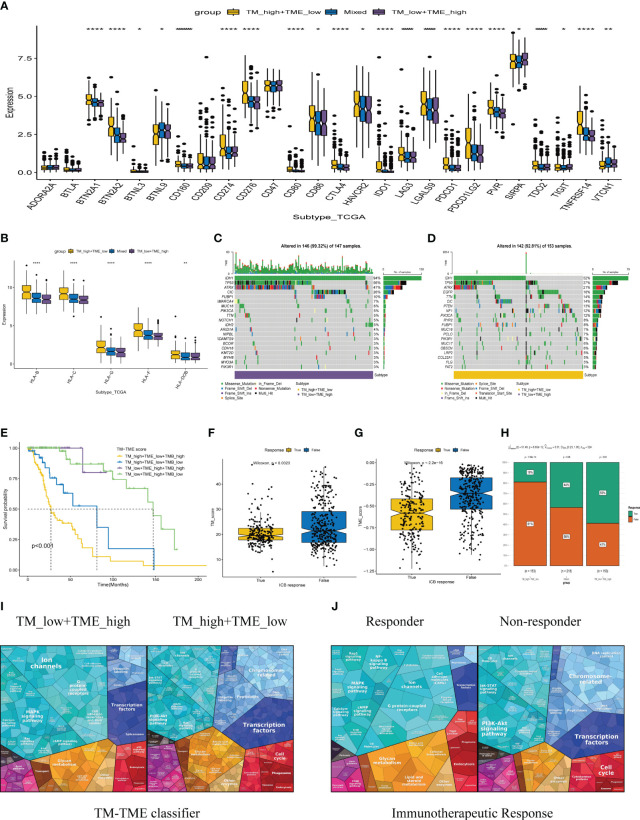
The TCGA-LGG cohort-related TM-TME classifier shows associations with immune checkpoints and somatic mutation. **(A)** Immune checkpoint genes with differential expression across various TM-TME classifier subgroups. **(B)** The differential expression levels of major histocompatibility complex (MHC) among TM-TME classifier subgroups. **(C, D)** Differences in mutations between the TM_H+TME_L subgroup and the TM_L+TME_H subgroup (the top 20 mutated genes). **(E)** KM-OS curves among four groups divided by TMB and TM-TME classifier. *p<0.05, **p<0.01, ****p<0.0001. **(F, G)** The TM and TME scores have different distributions among those who respond to immunotherapy and those who do not respond. **(H)** Comparison of immunotherapy responses across different TM-TME classifier groups in TCGA LGG cohort. **(I, J)** Functional analysis proteomaps of immunotherapy responders and non-responders in TM_H+TME_L, TM_L+TME_H subgroups. Polygons represent each KEGG pathway, with their sizes reflecting the protein ratio in each.

Immune checkpoint therapy confers long-term clinical benefits to patients in the field of oncology (44). Consequently, we investigated the expression profiles of crucial checkpoint genes and major histocompatibility complex (MHC) across distinct subgroups within the TM-TME paradigm. Notably, within the TCGA LGG cohort, significant variations in expression were observed for most checkpoint genes and MHC components (**Figures 9A, B**). Interestingly, the TM_H+TME_L subgroup exhibited elevated expression levels of MHCs and a majority of checkpoint genes, including BTN2A1, BTN2A2, CD274, CD276, CD86, CTLA4, among others.

The occurrence and accumulation of somatic mutations (SMs) span an individual’s lifetime, with a proposed theory suggesting that the progressive accumulation of genetic mutations contributes to the onset and progression of cancer (45). In addition, we explored the landscape of SMs across different subgroups within the TM-TME context. To illustrate this, **Figures 9C, D** presents a waterfall diagram showcasing the top 20 genes with the highest mutation frequencies in the TM_L+TME_H and TM_H+TME_L subgroups. In comparison to the TM_H+TME_L subgroup, the TM_L+TME_H subgroup exhibited a higher incidence of both gene mutations and mutations occurring in patients. The mutation rate in the TM_L+TME_H subgroup reached 99.32%, whereas the TM_H+TME_L subgroup had a mutation rate of 92.81%. Furthermore, distinct differences between the subgroups were observed in terms of higher frequencies of IDH1 mutations (94% in the TM_L+TME_H subgroup and 52% in the TM_H+TME_L subgroup), TP53 mutations (56% in the TM_L+TME_H subgroup and 37% in the TM_H+TME_L subgroup), and ATRX mutations (47% in the TM_L+TME_H subgroup and 21% in the TM_H+TME_L subgroup). Integrating the TM-TME classifier with tumor mutational burden (TMB) using the median TMB value in patients from the TCGA LGG cohort, we discovered a negative association between TMB and patients’ prognoses in the dataset (**Figure 9E**).

### 3.7 TM-TME classifier guided LGG therapy approaches

Immunotherapy-based strategies currently dominate as the prevailing therapeutic approaches for cancer. We employed TIDE to predict the response to immunotherapy in various subgroups of the tumor microenvironment (TM-TME). Notably, the responder group exhibited lower TM and TME scores in contrast to the non-responder group (**Figures 9F, G**). In specific subgroups, namely TM_L+TME_H, Mixed, and TM_H+TME_L, the response rates to immunotherapy were 59%, 44%, and 19%, respectively (**Figure 9H**). Of particular interest, the TM_L+TME_H subgroup demonstrated a significantly higher likelihood of benefiting from immunotherapy compared to the other two subgroups. To visually depict and differentiate the underlying processes among LGG patients in distinct groups, proteomaps were utilized (**Figures 9I, J**). Notably, the proteomaps of the TM_L+TME_H subgroup and responder groups exhibited a remarkably high level of similarity, while a similar observation was made between the TM_H+TME_L subgroup and non-responder groups. This indicates the effective characterization of the TME in LGG patients and the ability of the TM-TME classifier to predict the outcome of immunotherapy.

### 3.8 The expression levels of 17 prognostic risk genes

**Figure 10:**
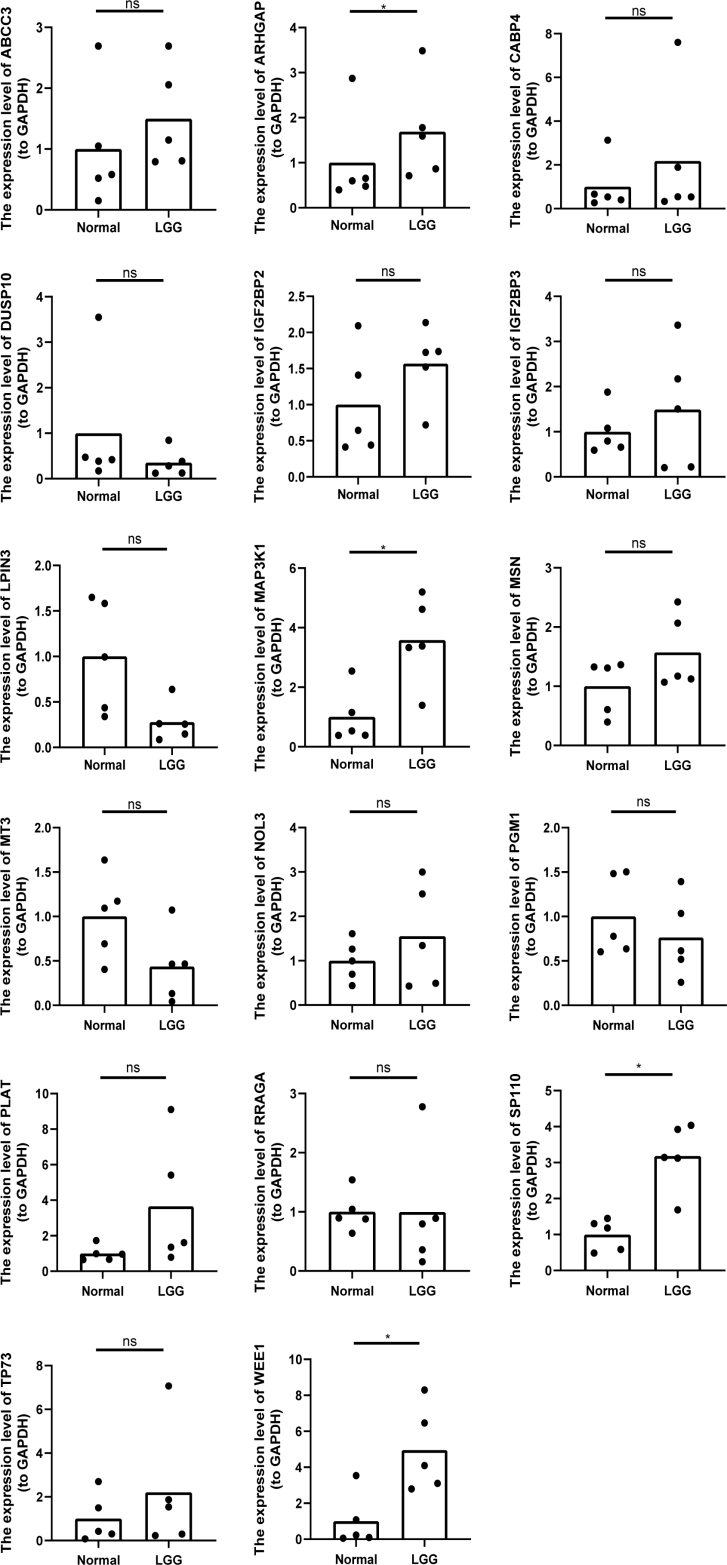
The relative expression levels of 17 genes (except GATA5) in five LGG tissues and the corresponding peritumoral tissues of patients. *p<0.05, ns represents no significant difference.

Using our bioinformatics analysis findings as a basis, we conducted additional investigations into the expression patterns of the 18 prognostic risk genes within five LGG tissues as well as their corresponding peritumoral tissues among patients. Employing qRT-PCR at the transcriptional level, we observed noteworthy disparities in the expression levels of four genes (ARHGAP, MAP3K1, SP110, and WEE1) between LGG and peritumoral tissues. However, no significant differences were detected in the expression levels of the remaining genes. Notably, GATA5 was undetectable in both LGG and peritumoral tissues as determined by qRT-PCR analysis (**Figure 10**).

### 3.9 The expression levels of WEE1 in LGG

**Figure 11:**
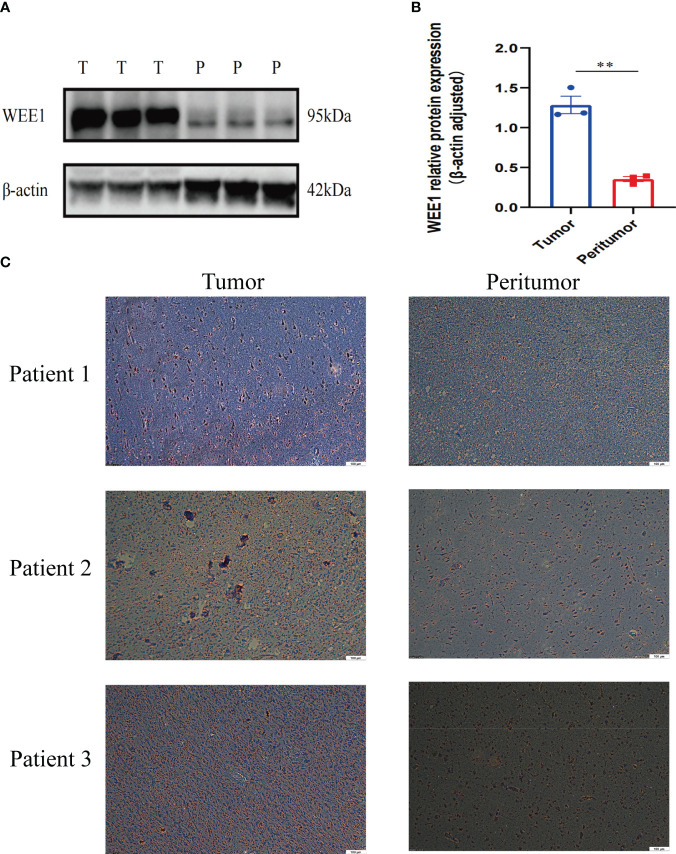
**(A)** WB for WEE1 in tumor tissues and peritumoral tissues from one patient. **(B)** The expression level of WEE1 in tumor tissue and peritumoral tissue. **(C)** IHC for WEE1 in paired tumor tissues and peritumoral tissue from three patients. **p<0.01.

**Figure 12:**
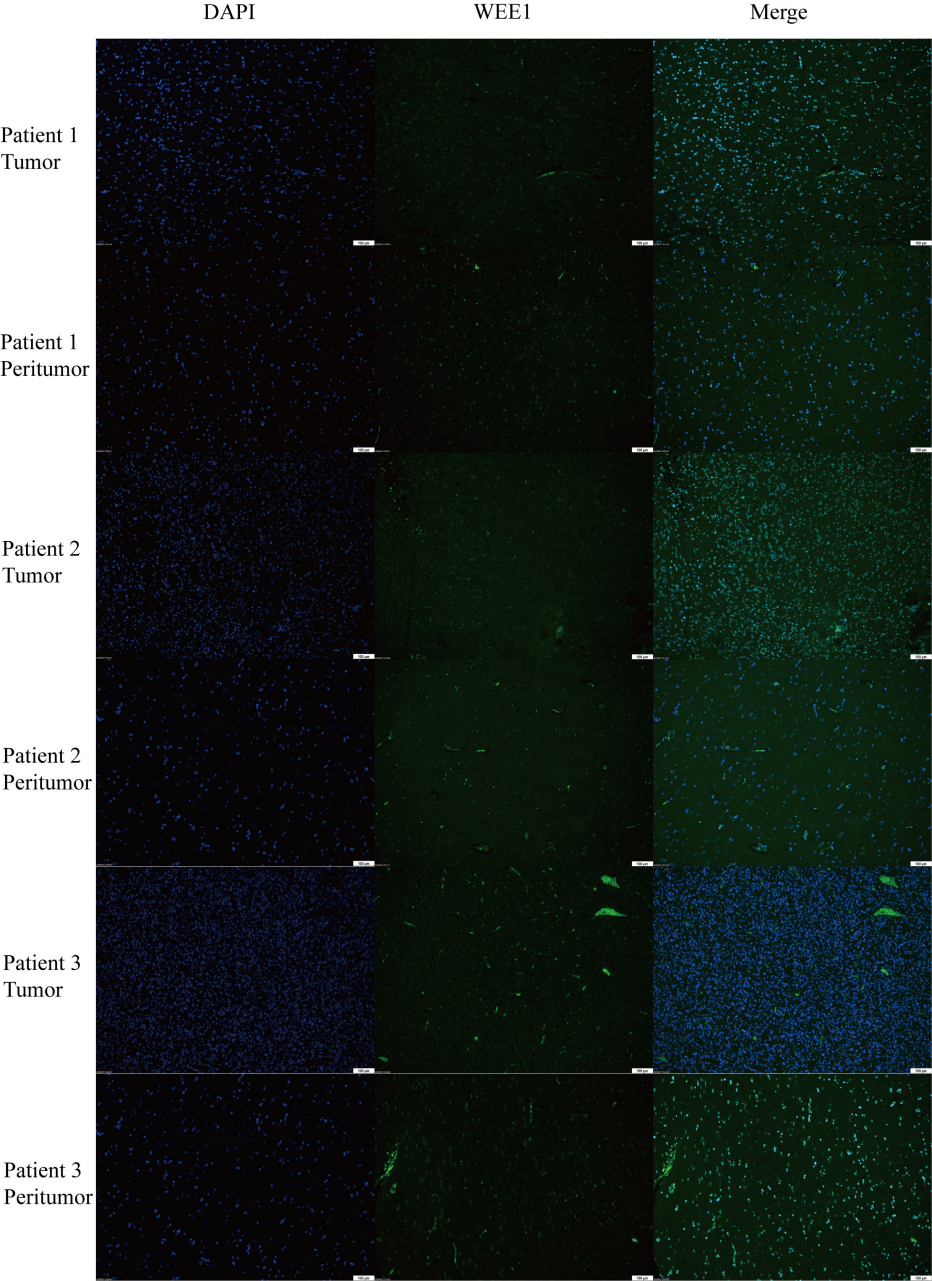
Immunofluorescence staining for WEE1 in paired tumor tissues and peritumoral tissue from three patients.

We conducted a random selection of a specific gene, WEE1, to investigate its protein expression patterns. Our analysis utilizing Western blotting (WB), immunohistochemistry (IHC), and immunofluorescence staining revealed a substantial elevation of WEE1 levels within the tumor tissues, in comparison to the corresponding adjacent tumor tissues. These findings strongly suggest that WEE1 holds great promise as a potential therapeutic target for glioma treatment (**Figures 11**, **12**).

## 4 Discussion

Telomeres have a crucial impact on the advancement of lower-grade glioma and the modulation of the tumor microenvironment (TME) (46). Limited investigations have explored the joint utilization of telomere and TME signatures to forecast prognosis and treatment response. The development of multi-omics has greatly improved the diagnostic and predictive accuracy of diseases (47, 48). In this novel investigation, we present the inaugural integration of telomere and TME signatures, resulting in the development of a TM-TME classifier capable of enhancing clinical categorization and refining treatment strategies.

In our study, we established the TM score utilizing the 18 genes associated with telomeres. Previous investigations have indicated the upregulation of ABCC3 (ATP binding cassette subfamily C member 3) in glioma patients (49). The hypomethylation of ABCC3 has been observed in LGG patients with epilepsy, which is associated with unfavorable prognoses (50). Furthermore, the methylation of GATA5 (GATA binding protein 5) has been identified in 27.8% of glioblastoma patients and is strongly correlated with poor outcomes in primary glioblastoma cases (51). Activation of the PI3K/Akt signaling pathway by IGF2BP2 (also known as Imp2, Insulin-like growth factor 2 mRNA-binding protein 2) promotes glioma progression, while its inhibition sensitizes glioma cells to temozolomide treatment (52). Glioma cell proliferation, invasion, and tumor propagation have been found to be facilitated by IGF2BP3, as observed by Jin et al. (53). Extensive research has shown that MAP3K1 plays a crucial role in cell migration, growth, and apoptosis, and its dysregulation is closely associated with adverse outcomes in various malignancies, including glioma (54–57). Some genes, such as CABP4, DUSP10, LPIN3, were reported for the first time to be associated with glioma.

The immune system plays a crucial role in cancer development and the advancement of immunotherapy (58). Extensive research is currently being conducted on exosome-based immunotherapy (59, 60). It is noteworthy that metabolic molecules exert a substantial influence on the immune environment, thereby impacting disease progression (61–63). Moreover, the equilibrium of cytokines holds significant sway over the advancement of diseases (64). To better understand the role of immune cells in lower-grade glioma, subsequently, we developed the TME score based on five TME cells: eosinophils, activated mast cells, monocytes, plasma cells, and activated NK cells. These cells are all associated with a favorable prognosis in LGG patients. Eosinophils, derived from myeloid progenitors, have been found to correlate with improved prognoses in several solid tumors, including glioma, colon cancer, and lung cancer, due to their infiltration and degranulation (65–67). Mast cells suppress the signal transducer and activator of transcription 3 (STAT3) pathway by downregulating glycogen synthase kinase 3β (GSK3β), thereby diminishing the proliferation, migration, and invasion of glioma cells (68). Monocytes are found in the spleen, blood, and bone marrow (69). They encompass three subtypes: monocytes, repolarized monocytes, and monocytes that differentiate into macrophages (70). Wang et al. observed a decrease in invasive monocyte counts and a subtype-dependent increase in macrophage/microglia numbers during glioma recurrence, as determined by a gene signature associated with the TME (71). The expression of B cell and plasma cell signature genes has been positively correlated with overall survival (OS) in patients with pancreatic cancer, melanoma, and lung adenocarcinoma. However, elevated expression levels of these genes have been associated with poorer clinical outcomes in patients with glioblastoma and clear cell renal cell carcinoma (72–74).

The TM-TME classifier was established based on the TM and TME scores. Analysis using Gene Ontology (GO) indicated that the TM_L+TME_H subgroup is associated with neuronal system function and the modulation of chemical synaptic transmission. Notably, studies have demonstrated that gliomas can perturb neuronal plasticity and development within the TME, leading to aberrant neuronal connections with tumor cells through neuronal glioma synapses (75, 76). On the other hand, the TM_H+TME_L subgroup exhibits a stronger correlation with cell cycle regulation and DNA metabolic processes. Previous research by Roth et al. has highlighted the role of glycosylation, lipid metabolism, and carbohydrate metabolism in promoting tumor malignancy, thereby identifying novel therapeutic targets (77). Consistently, our findings reveal a poorer prognosis in the TM_H+TME_L subgroup.

Interestingly, patients assigned to the TM_L+TME_H subgroup displayed the most favorable prognosis, which was positively associated with higher tumor mutational burden (TMB). TMB has emerged as a potential biomarker for predicting the efficacy of immune checkpoint blockade (ICB) therapy. Hence, the TM_L+TME_H subgroup exhibits a higher rate of immune response to treatment. In contrast, the TM_H+TME_L subgroup exhibited the poorest prognosis, accompanied by the lowest immune response rate. These results are in line with previous perspectives on the relationship between TMB and immunotherapy (78).

In brief, we have delineated the combined molecular profiles of telomeres and immune cells in the tumor microenvironment (TME). This comprehensive analysis provides a fresh perspective on the classification of low-grade gliomas (LGGs) and offers accurate prognostic predictions as well as insights into the effectiveness of immune-based therapies. Nonetheless, it is important to acknowledge the limitations of our study. Firstly, the sample size utilized in our investigation was relatively small, which may impact the generalizability of our findings. Secondly, the inclusion of 18 telomere-associated genes in our analysis poses a significant challenge in terms of experimental validation. Besides, the migratory capacity and drug resistance of tumor cells are closely associated with adverse prognosis and recurrence, thus necessitating further experimental elucidation of underlying mechanisms (79, 80). Lastly, the absence of an LGG-specific dataset for assessing the performance of our classifier, despite its validation in the TCGA and CCGA cohorts, is a notable constraint.

## Data availability statement

The datasets presented in this study can be found in online repositories. The names of the repository/repositories and accession number(s) can be found within the article/**Supplementary Material**.

## Ethics statement

The studies involving human participants were reviewed and approved by the Ethical Review Committee of Beijing Tiantan Hospital. The patients/participants provided their written informed consent to participate in this study.

## Author contributions

XH and JG conceived the study and collected and analyzed the data. XH wrote the manuscript. JG, YZ, LA provided technical guidance and experimental guidance. XH, ZY, KF, XG, BH, XL, YO, BC contributed to data collection, analysis and interpretation, and manuscript writing. All authors contributed to the article and approved the submitted version.

## Supplementary material

Supplementary Figure 1 The expression analysis of marker genes using single cell analysis. **(A, B)** t-SNE plot of cells from two datasets, respectively, with each color and number coded to indicate the associated cell types. **(C, D)** DotPlots show the expression levels of marker genes in each cluster of two datasets, respectively.

Supplementary Figure 2 WGCNA analysis and construction of interactive network. GO enrichment analysis was applied to each MCODE network. The same color nodes represent an interactive network and perform similar biological functions. **(A)** Clustering of sample data to detect outliers. (cutHeight =160) **(B)** Analysis of the scale-free fit index (left) and the mean connectivity (right) for various soft-thresholding power value. **(C)** Dendrogram of 5000 selected genes clustered based on a dissimilarity measure (1-TOM) together with assigned module colors. **(D)** Seven MCODE components were constructed with the screened hub genes in TM_high+TME_low subgroup. **(E)** Each interactive network with different colors own different score values in TM_high+TME_low subgroup. **(F)** Eleven MCODE components were constructed with the screened hub genes in TM_low+TME_high subgroup. **(G)** Each interactive network with different colors owns different score values in TM_low+TME_high subgroup.

Supplementary Figure 3 Kaplan-Meier overall survival curves of TM-TME classifier in diverse LGG clinical subtypes in TCGA LGG cohort **(A–J)**.
