# Supplementary figures and images for: The combined signatures of telomere and immune cell landscape provide a prognostic and therapeutic biomarker in glioma
Source: Front Immunol. 2023 Aug 17;14:1220100. doi: 10.3389/fimmu.2023.1220100 (PMC10470026; doi:10.3389/fimmu.2023.1220100)

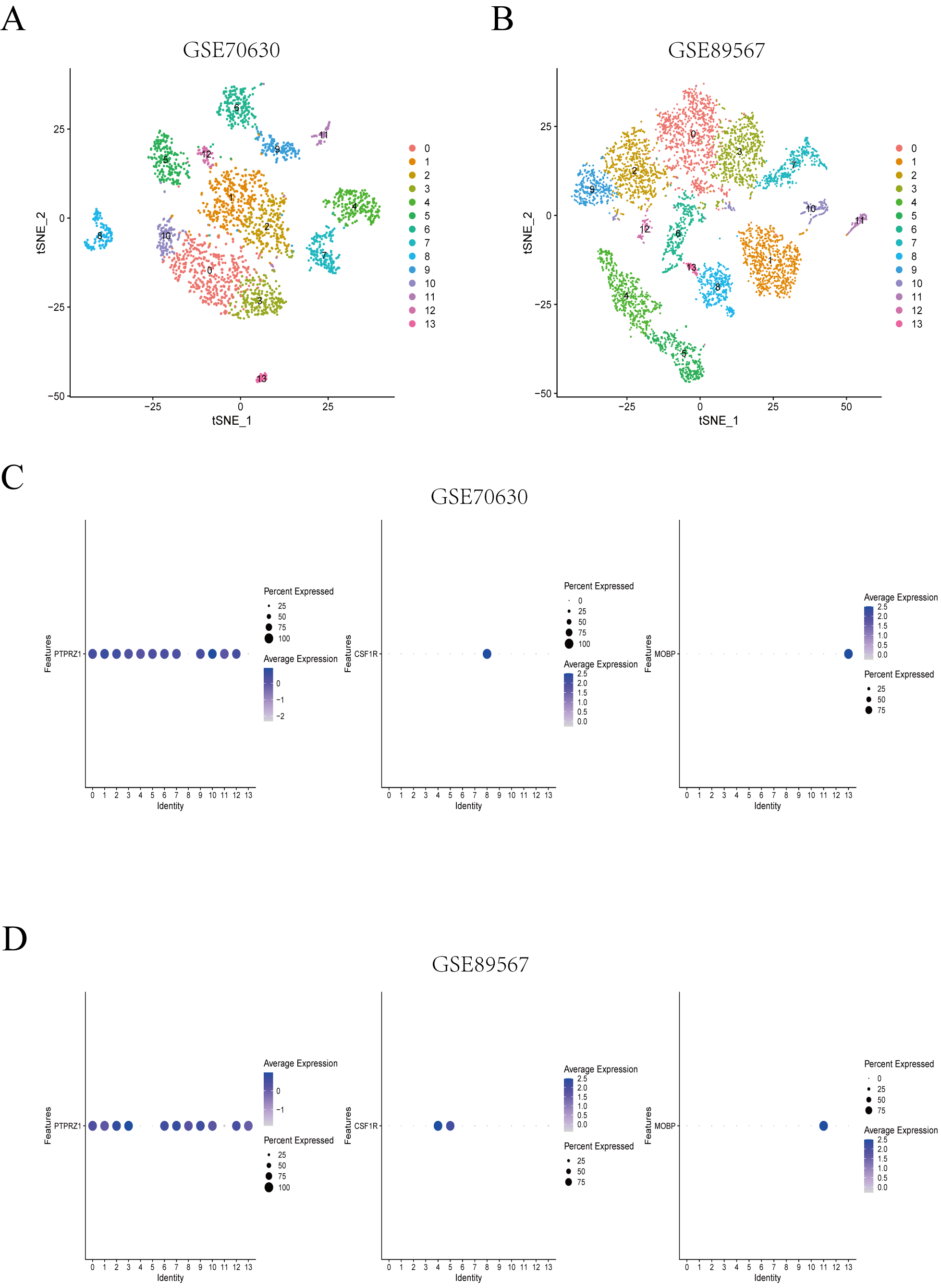

Supplement: Supplementary Figure 1 — The expression analysis of marker genes using single cell analysis. (A, B) t-SNE plot of cells from two datasets, respectively, with each color and number coded to indicate the associated cell types. (C, D) DotPlots show the expression levels of marker genes in each cluster of two datasets, respectively. [file Image_1.tif]

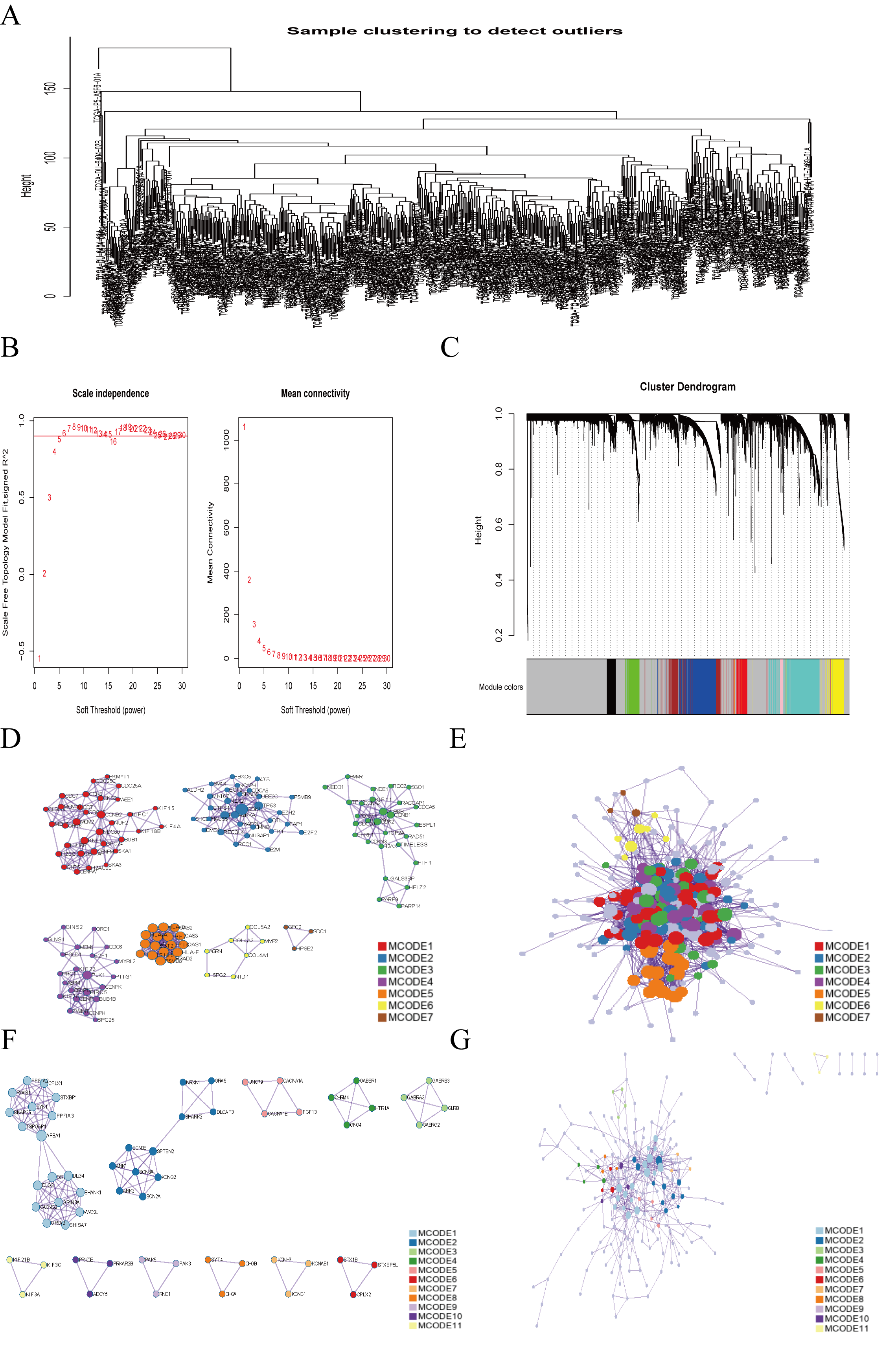

Supplement: Supplementary Figure 2 — WGCNA analysis and construction of interactive network. GO enrichment analysis was applied to each MCODE network. The same color nodes represent an interactive network and perform similar biological functions. (A) Clustering of sample data to detect outliers. (cutHeight =160) (B) Analysis of the scale-free fit index (left) and the mean connectivity (right) for various soft-thresholding power value. (C) Dendrogram of 5000 selected genes clustered based on a dissimilarity measure (1-TOM) together with assigned module colors. (D) Seven MCODE components were constructed with the screened hub genes in TM_high+TME_low subgroup. (E) Each interactive network with different colors own different score values in TM_high+TME_low subgroup. (F) Eleven MCODE components were constructed with the screened hub genes in TM_low+TME_high subgroup. (G) Each interactive network with different colors owns different score values in TM_low+TME_high subgroup. [file Image_2.tif]

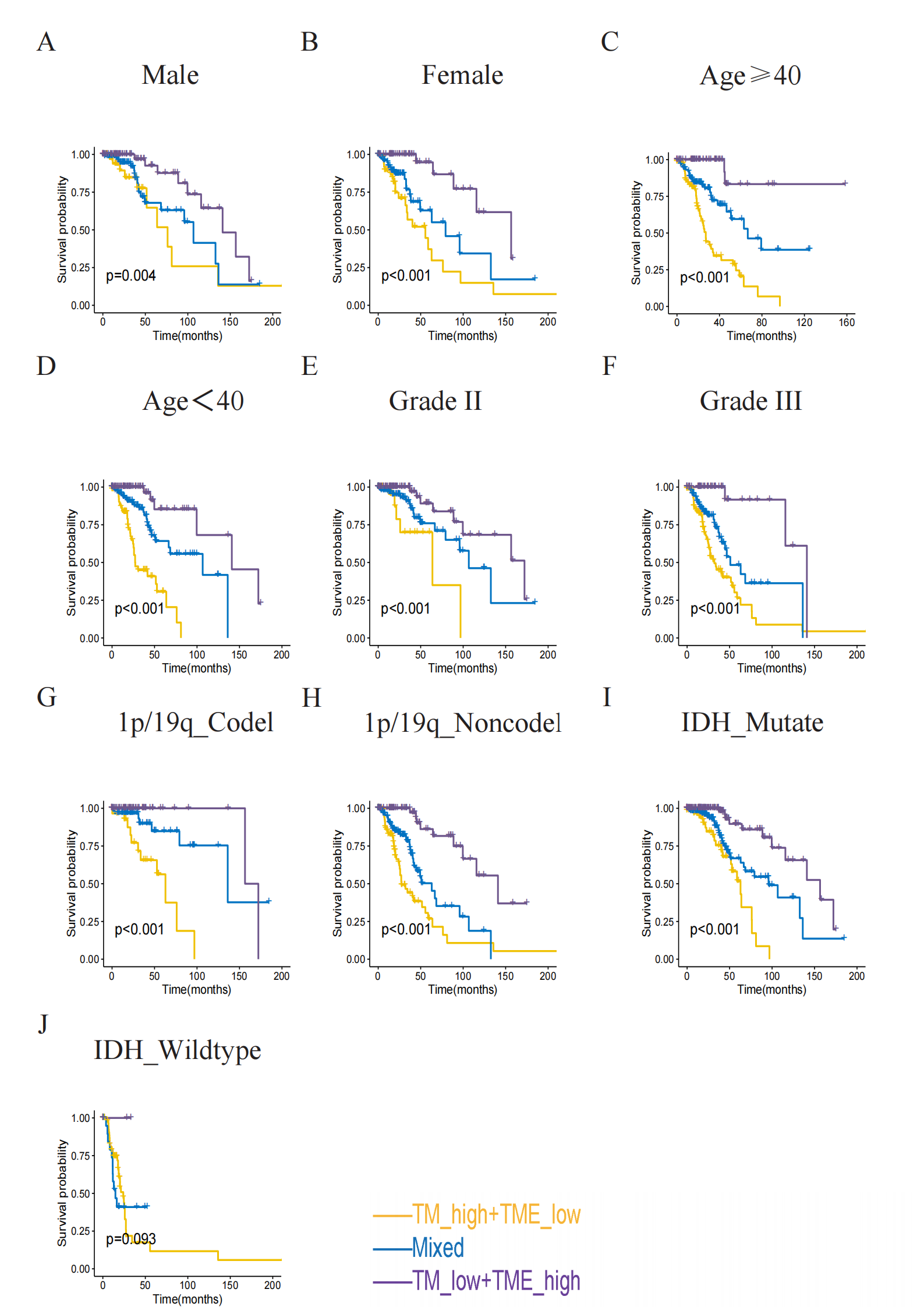

Supplement: Supplementary Figure 3 — Kaplan-Meier overall survival curves of TM-TME classifier in diverse LGG clinical subtypes in TCGA LGG cohort (A–J). [file Image_3.tif]
